# Sexually Dimorphic Accumulation of Persistent Organic Pollutants in Fetuses

**DOI:** 10.3389/ftox.2022.909307

**Published:** 2022-05-17

**Authors:** Richelle D. Björvang, Linn Salto Mamsen

**Affiliations:** ^1^ Department of Women’s and Children’s Health, Uppsala University, Uppsala, Sweden; ^2^ Division of Obstetrics and Gynecology, Department of Clinical Science, Intervention and Technology, Karolinska Institutet and Karolinska University Hospital Huddinge, Stockholm, Sweden; ^3^ Laboratory of Reproductive Biology, The Juliane Marie Centre for Women, Children and Reproduction, University Hospital of Copenhagen, University of Copenhagen, Copenhagen, Denmark

**Keywords:** human fetal exposure, persisting organic pollutants, sex differences, chemical transfer, PCB, OCP, PBDE, PFAS

## Abstract

Living in an industrialized era, we are exposed to man-made chemicals including persistent organic pollutants (POPs). Previous studies have shown associations of POP exposure with adverse outcomes in humans, wildlife, and the environment, making it a global concern. Exposure during sensitive windows of susceptibility such as fetal development is of particular concern because of the potential increased risk of developing diseases in childhood and adulthood. However, there are limited studies on the sexual dimorphism of POP accumulation during the prenatal period. In this mini-review, we focus on differences in POP concentrations in the placenta and fetal tissues between males and females. We also show the sexually dimorphic adverse outcomes of prenatal exposure to POPs. Overall, our summary shows that males may accumulate higher concentrations of POPs in the placenta and fetal tissues compared to females, although studies are sparse and inconsistent. In addition, there are differences in adverse health outcomes associated to prenatal POP exposure according to sex. Hence, we highly urge researchers investigating the health effects of POP exposure to consider sexual dimorphism in their studies.

## Introduction

Our modern, industrialized society has developed countless improvements for everyday life. Although these developments come with many benefits, they can also cause adverse health effects in humans and wildlife. One important example is exposure to man-made industrial chemicals such as persistent organic pollutants (POPs), which is now ubiquitous in day-to-day life. POPs are halogenated organic chemicals, which means that they are carbon-based with either chlorine, fluorine or bromine attached to the carbon. Due to their structure and high stability, they are highly resistant to degradation. Their half-lives range from one to 30 years in humans (Trudel et al., 2011; Bu et al., 2015; Li et al., 2018). They are persistent, toxic and bioaccumulate in living organisms including humans. Some are volatile at certain temperatures, making them widely distributed throughout the environment, and can be found even in areas thousands of kilometers away from their sources (Stockholm Convention, 2008b).

These man-made chemicals have been found to disrupt endocrine activity and are thus named endocrine-disrupting chemicals (EDCs). Studies of humans, wildlife, multiple experimental animals, and epidemiological cohorts show associations between EDC exposure and adverse health effects, making this a global health concern (Bergman et al., 2013). These derived effects of EDC exposure have a negative impact not only on the health and well-being of humans and wildlife but also to the economy. A recent estimate suggested that the European countries alone spend 157 billion euros a year to treat health disorders derived from EDC exposure (Trasande et al., 2015). This estimate included only three health outcomes (reproductive health, neurodevelopmental disorders, and metabolic disorders) and were based on a limited selection of EDCs (brominated flame retardants, pesticides, and phthalates and bisphenol A), implying that the true cost of EDC exposure is expected to be even larger. Therefore, EDC exposure is likely to contribute substantially to burden of disease and costs.

The primary route of exposure to POPs in humans is through ingestion, inhalation and absorption (Bergman et al., 2013; Gore et al., 2015). Further exposure occurs through placental transfer from mother to the fetus (Mamsen et al., 2017, 2019; Björvang et al., 2021), and after birth through breastfeeding (Krysiak-Baltyn et al., 2010). Because POPs are found almost everywhere, exposure of the next generation starts even before conception. Studies have shown that human oocytes are extensively exposed to mixtures of these chemicals (Björvang and Damdimopoulou, 2020; Björvang et al., 2021; Lefebvre et al., 2021). Several studies have also found POPs to have a negative effect on human spermatogenesis, though the pathophysiological mechanisms are not fully elucidated (Toft et al., 2012; Sharma et al., 2020).

This mini-review focuses on a subgroup of POPs including organochlorine pesticides (OCPs), polychlorinated biphenyls (PCBs), polybrominated diphenyl ethers (PBDEs), and perfluoroalkyl substances (PFASs), which are still widely distributed in our environment. OCPs, PCBs, and PBDEs are lipophilic and deposit to adipose tissue (Mustieles and Arrebola, 2020) while PFASs are amphiphilic and predominantly bind to proteins (Forsthuber et al., 2020). The OCPs, PCBs, and PBDEs have been banned for several decades while the PFASs has just been recently regulated (Stockholm Convention, 2008a). Nonetheless, they are still present in the environment due to their high resistance to degradation as well as unintentional production as industrial by-products.

The negative impact of POPs on health is increasingly well-documented and it is becoming clear that exposure to POPs during critical windows of susceptibility such as fetal development can have long-lasting consequences for the exposed individual (Bergman et al., 2013; Gore et al., 2015). Animal studies have revealed that prenatal exposure to POPs are associated with reduced postnatal survival, low birth weight, epigenetic alterations, disrupted thyroid function, birth defects including cleft palate, anasarca, and heart disorders, and compromised fertility (Thibodeaux et al., 2003; Lau et al., 2004, 2006; Yu et al., 2009; Guo et al., 2014; Negri et al., 2017). Human prenatal exposure to POPs have been associated with reduced birth weight (Fei et al., 2007; Maisonet et al., 2012; Johnson et al., 2014; Callan et al., 2016; Lauritzen et al., 2016), though reports are inconsistent (Olsen et al., 2009; Bach et al., 2015; Yang et al., 2021). In addition, an increased risk for congenital cerebral palsy, retarded lung maturation, and thyroid dysregulation have been reported (Maervoet et al., 2007; Abdelouahab et al., 2013; Liew et al., 2014; Luo et al., 2017; Sørli et al., 2020). While studies have investigated associations between prenatal exposure and adverse health outcomes, only few studies considered potential sex-specific differences. In this mini-review, we focus on the sexual dimorphism of prenatal POP exposure.

### Human Male Fetuses May Be More Exposed to POPs Than Female Fetuses

**FIGURE 1:**
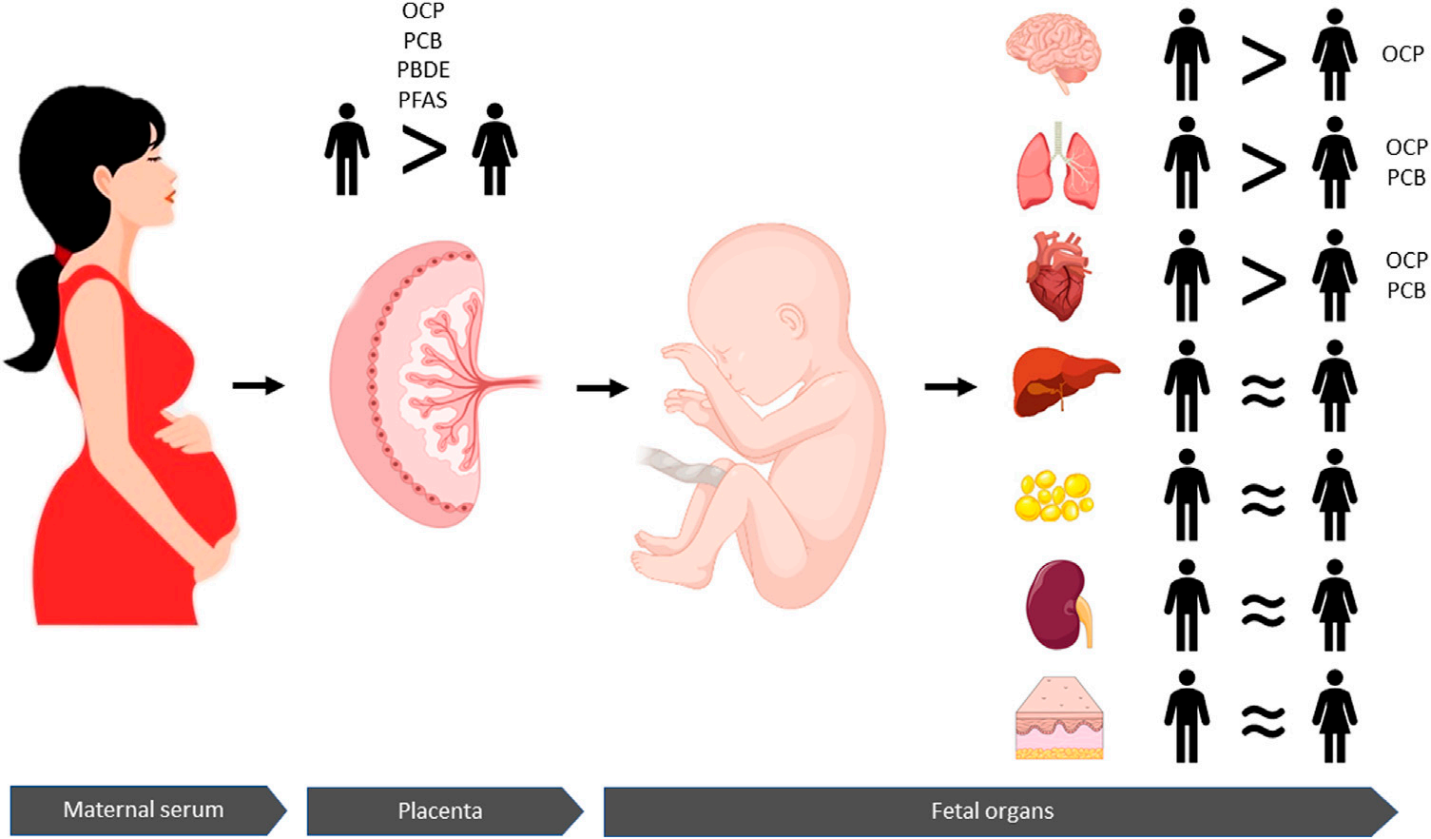
Persistent organic pollutants (POPs) are transferred from the maternal circulation *via* the placenta to the fetus and accumulate in different fetal organs. Concentrations of OCPs, PCBs, PBDEs, and PFASs are higher in human placentas with male fetuses compared to placentas with female fetuses. Moreover, this sexually dimorphic patten is reflected in the fetal organs where higher concentrations of OCPs accumulate in males' in brain, lung and hearts tissues than those of females. Concentrations of PCBs are also higher in male lungs and heart tissues than females. Similar concentrations of POPs are found in liver, fat, kidney, and skin tissues from male and female fetuses. Taken together, these findings suggest that male fetuses are overall more exposed to POPs than female fetuses, which may be associated with sexual dimorphic risks.

Since it is not possible to acquire fetal tissues in birth cohorts, there are only a limited number of studies with the unique opportunity to analyze the actual chemical concentrations in human fetal tissues. The available data are from fetal tissues obtained either from elective pregnancy terminations or stillbirths (Curley et al., 1969; Nishimura et al., 1977; Schecter et al., 2006; Doucet et al., 2009; Pusiol et al., 2016; Mamsen et al., 2017, 2019; Zota et al., 2018; Björvang et al., 2021). Among these nine studies, only three investigated the sexual dimorphism of POP exposure (Nishimura et al., 1977; Mamsen et al., 2019; Björvang et al., 2021) while Zota and colleagues (2018) only adjusted for fetal sex when looking into the association between fetal liver PBDEs and fetal cytochrome P450 gene expression. Nishimura et al. (1977) did not find any relationship between fetal sex and OCPs and PCBs in the fetal brain, heart, liver, kidneys, and skin. Similarly, Mamsen et al. (2019) found no association between fetal sex and PFASs in the fetal tissues including central nervous system, heart, lung, liver, and adipose tissue. On the other hand, Björvang et al. (2021) found higher concentrations of OCPs and PCBs in the male fetal brain, heart, and lung. In general, different subgroups of POPs accumulate to different fetal organs. The highest fetal PFAS concentrations were detected in liver and lung tissues, whereas the OCPs and PCBs primarily accumulated to fetal adipose tissue (Mamsen et al. (2019); Björvang et al., 2021). However, more studies are needed in relation to sexual dimorphism. Taken together, male sex may be associated with higher fetal concentrations of some POPs, though data are sparse and inconsistent (Figure 1).

### Higher Levels of POPs Accumulate in Placentas With Male Fetuses Than Placentas With Female Fetuses

Prenatal exposure to chemicals is commonly estimated through surrogate matrices such as the placenta, among others. Studies have detected higher concentrations of POPs including OCPs, PBDEs, and PCBs in human placental tissues with male newborns compared to placentas with female newborns (Leonetti et al., 2016; Kim et al., 2019). Higher concentrations of OCPs and PCBs have been reported in human placentas with male fetuses in second and third trimester of pregnancy (Björvang et al., 2021) and higher placental PFASs concentrations can be detected in pregnancies with male fetuses already during first trimester (Mamsen et al., 2019), suggesting that the sex dimorphic chemical transfer is established very early in fetal life and throughout pregnancy (Figure 1).

### Sexually Dimorphic Placental Development

Rodent models have demonstrated a sexually dimorphic development of the placenta, with a slower placental development and more protective adaptive responses to stressful environments in females compared to males (Kalisch-Smith et al., 2017; Saoi et al., 2020). Transcriptome analysis of human first- and second trimester placentas confirm different expression patterns between the sexes (Gonzalez et al., 2018; Braun et al., 2021), which can also be detected in term placentas (Sood et al., 2006). When the intrauterine environment is disturbed in rodents and humans, placentas with male fetuses consume more energy to accelerate its own growth as well as the fetus’, accelerating epigenetic aging at the expense of adaptability and plasticity (Tekola-Ayele et al., 2019; Yu et al., 2021). In contrast, placentas with female fetuses respond to disturbances with protective mechanisms such as slowing down growth and metabolism to ensure fetal survival (Miller et al., 2020; Phuthong et al., 2020; Saoi et al., 2020; Weinheimer et al., 2020). These different growth strategies in response to disturbances may place male fetuses at a greater risk when exposed to POPs or other disturbing pollutants. In addition, sexually dimorphic placental function has also been reported in humans, which may facilitate a skewed accumulation of chemicals between the sexes (Rosenfeld, 2015). Pregnancies with female fetuses have higher placental vascular resistance compared to that of pregnancies with male fetuses (Widnes et al., 2017), which may affect the blood flow to the fetus and thereby, support a different chemical transfer between sexes. Moreover, epigenetic patterns in the placenta differed between the sexes (Martin et al., 2017), which may also explain the diverging susceptibility to chemicals and health outcomes. For example, maternal exposure to OCPs, PCBs, and PBDEs have been associated with sex-dimorphic epigenetic changes in placental genes involved in the placental transfer of thyroid hormones (Kim et al., 2019). The human fetal metabolizing machinery has also been evaluated in relation to maternal use of over-the-counter analgesics and found that some enzymes involved in pharmacokinetic and pharmacodynamic pathways are significantly affected by fetal sex and gestational age (Zafeiri and Fowler, 2021). Taken together, these reports implies that the higher accumulation of chemicals observed in male fetuses than female fetuses may be caused by different placental development, function, epigenetics, and metabolism between the sexes.

### Adverse Outcomes Differ According to Sex

Sexually dimorphic adverse outcomes of PFAS exposure have been described in rodents, where prenatal exposure to PFOA significantly affected fetal growth and development in males but to a much lesser extent in females (Nakayama et al., 2005; Negri et al., 2017). The higher sensitivity of male fetuses may be related to a less efficient elimination of PFOA in males, which have been observed in rodents (Vanden Heuvel et al., 1991). In humans, *in utero* exposure to POPs has been associated with reduced birth weight in some studies, although results are inconsistent (Olsen et al., 2009; Tan et al., 2009; Govarts et al., 2012; Bach et al., 2015; Casas et al., 2015; Lauritzen et al., 2017). One study found the reduced birth weight to be more pronounced in male offspring (Lauritzen et al., 2017) while another study found the correlation only in female offspring (Washino et al., 2009). On the other hand, Covarts and colleagues (2012) did not find any sex-specific effects.

Prenatal exposure to some OCPs has been associated with higher body mass index (BMI) in girls up to two years after birth (Mendez et al., 2011; Valvi et al., 2014; Coker et al., 2018; Yang et al., 2021), implying that prenatal exposure may cause physiological changes predisposing to later weight gain. In addition, *in utero* exposure to PFAS has been positively associated with BMI in females at 20 years of age (Halldorsson et al., 2012). Exposure to EDCs has been associated with an increased obesity risk, which may be due to disturbance in the regulation of endocrine hormones or the hypothalamic-pituitary-adrenal axis that regulate homeostatic mechanisms important to weight control (Grün and Blumberg, 2009; Tang-Péronard et al., 2011).

Prenatal exposure to OCPs and PBDEs have been associated with reduced maternal supply of thyroid hormones to the fetus, which may affect the thyroid hormone balance in newborns (Maervoet et al., 2007; Abdelouahab et al., 2013; Luo et al., 2017; Krönke et al., 2021). Prenatal exposure to PCBs were positively associated with the level of thyroid hormones in female children 6 months after birth, while OCPs were negatively associated with the level of thyroid hormones of male children 1 year after birth (Krönke et al., 2021).

## Conclusion

POPs accumulate at higher concentrations in human male fetuses and their placentas than in female fetuses and their placentas. This may be caused by a sexually dimorphic genetic and epigenetic regulation, placental resistance, and protective strategies to external stress. The sexual dimorphic fetal accumulation may be caused by differences in placental transfer and by differences in fetal pharmacokinetic, and endocrine milieu. Prenatal exposure to POPs has been associated with adverse health outcomes, though sex-specific effects are sparse and inconsistent. Most exposure studies do not monitor potential sex dimorphic outcomes, which leave us with a research gap that merits consideration and highlight the need for more studies including fetal sex as a potential covariate.

## References

[B1] Abdelouahab N. Langlois M.-F. Lavoie L. Corbin F. Pasquier J.-C. Takser L. (2013). Maternal and Cord-Blood Thyroid Hormone Levels and Exposure to Polybrominated Diphenyl Ethers and Polychlorinated Biphenyls during Early Pregnancy. Am. J. Epidemiol. 178, 701–713. 10.1093/AJE/KWT141 23924579

[B2] Bach C. C. Bech B. H. Brix N. Nohr E. A. Bonde J. P. E. Henriksen T. B. (2015). Perfluoroalkyl and Polyfluoroalkyl Substances and Human Fetal Growth: A Systematic Review. Crit. Rev. Toxicol. 45, 53–67. 10.3109/10408444.2014.952400 25372700

[B3] Bergman Å. Heindel J. Jobling S. Kidd K. Zoeller R. T. (2012). State-of-the-science of Endocrine Disrupting Chemicals, 2012. Toxicol. Lett. 211, S3. 10.1016/j.toxlet.2012.03.020

[B4] Björvang R. D. Damdimopoulou P. (2020). Persistent Environmental Endocrine-Disrupting Chemicals in Ovarian Follicular Fluid and *In Vitro* Fertilization Treatment Outcome in Women. Upsala J. Med. Sci. Med. Sci. 125, 85–94. 10.1080/03009734.2020.1727073 PMC7721012 32093529

[B5] Björvang R. D. Vinnars M.-T. Papadogiannakis N. Gidlöf S. Mamsen L. S. Mucs D. (2021). Mixtures of Persistent Organic Pollutants Are Found in Vital Organs of Late Gestation Human Fetuses. Chemosphere 283, 131125. 10.1016/j.chemosphere.2021.131125 34467953

[B6] Braun A. E. Muench K. L. Robinson B. G. Wang A. Palmer T. D. Winn V. D. (2021). Examining Sex Differences in the Human Placental Transcriptome during the First Fetal Androgen Peak. Reprod. Sci. 28, 801–818. 10.1007/S43032-020-00355-8 33150487

[B7] Bu Q. MacLeod M. Wong F. Toms L.-M. L. Mueller J. F. Yu G. (2015). Historical Intake and Elimination of Polychlorinated Biphenyls and Organochlorine Pesticides by the Australian Population Reconstructed from Biomonitoring Data. Environ. Int. 74, 82–88. 10.1016/j.envint.2014.09.014 25454223

[B8] Callan A. C. Hinwood A. L. Heyworth J. Phi D. T. Odland J. Ø. (2016). Sex Specific Influence on the Relationship between Maternal Exposures to Persistent Chemicals and Birth Outcomes. Int. J. Hyg. Environ. Health 219, 734–741. 10.1016/J.IJHEH.2016.09.018 27720132

[B9] Casas M. Nieuwenhuijsen M. Martínez D. Ballester F. Basagaña X. Basterrechea M. (2015). Prenatal Exposure to PCB-153, P,p′-DDE and Birth Outcomes in 9000 Mother-Child Pairs: Exposure-Response Relationship and Effect Modifiers. Environ. Int. 74, 23–31. 10.1016/J.ENVINT.2014.09.013 25314142

[B11] Coker E. Chevrier J. Rauch S. Bradman A. Obida M. Crause M. (2018). Association between Prenatal Exposure to Multiple Insecticides and Child Body Weight and Body Composition in the VHEMBE South African Birth Cohort. Environ. Int. 113, 122–132. 10.1016/J.ENVINT.2018.01.016 29421401 PMC5866210

[B12] Curley A. Copeland M. F. Kimbrough R. D. (1969). Chlorinated Hydrocarbon Insecticides in Organs of Stillborn and Blood of Newborn Babies. Archives Environ. Health Int. J. 19, 628–632. 10.1080/00039896.1969.10666901 4187028

[B13] Doucet J. Tague B. Arnold D. L. Cooke G. M. Hayward S. Goodyer C. G. (2009). Persistent Organic Pollutant Residues in Human Fetal Liver and Placenta from Greater Montreal, Quebec: A Longitudinal Study from 1998 through 2006. Environ. Health Perspect. 117, 605–610. 10.1289/ehp.0800205 19440500 PMC2679605

[B14] Fei C. McLaughlin J. K. Tarone R. E. Olsen J. (2007). Perfluorinated Chemicals and Fetal Growth: A Study within the Danish National Birth Cohort. Environ. Health Perspect. 115, 1677–1682. 10.1289/ehp.10506 18008003 PMC2072850

[B15] Forsthuber M. Kaiser A. M. Granitzer S. Hassl I. Hengstschläger M. Stangl H. (2020). Albumin Is the Major Carrier Protein for PFOS, PFOA, PFHxS, PFNA and PFDA in Human Plasma. Environ. Int. 137, 105324. 10.1016/j.envint.2019.105324 32109724

[B16] Gonzalez T. L. Sun T. Koeppel A. F. Lee B. Wang E. T. Farber C. R. (2018). Sex Differences in the Late First Trimester Human Placenta Transcriptome. Biol. Sex. Differ. 9. 165. 10.1186/S13293-018-0165-Y PMC5769539 29335024

[B17] Gore A. C. Chappell V. A. Fenton S. E. Flaws J. A. Nadal A. Prins G. S. (2015). EDC-2: The Endocrine Society's Second Scientific Statement on Endocrine-Disrupting Chemicals. Endocr. Rev. 36, E1–E150. 10.1210/er.2015-109310.1210/er.2015-1010 26544531 PMC4702494

[B18] Govarts E. Nieuwenhuijsen M. Schoeters G. Ballester F. Bloemen K. de Boer M. (2012). Birth Weight and Prenatal Exposure to Polychlorinated Biphenyls (PCBs) and Dichlorodiphenyldichloroethylene (DDE): a Meta-Analysis within 12 European Birth Cohorts. Environ. Health Perspect. 120, 162–170. 10.1289/EHP.1103767 21997443 PMC3279442

[B19] Grün F. Blumberg B. (2009). Endocrine Disrupters as Obesogens. Mol. Cell. Endocrinol. 304, 19–29. 10.1016/J.MCE.2009.02.018 19433244 PMC2713042

[B20] Guo Y. Hai Y. Gong Y. Li Z. He Z. (2014). Characterization, Isolation, and Culture of Mouse and Human Spermatogonial Stem Cells. J. Cell. Physiol. 229, 407–413. 10.1002/jcp.24471 24114612

[B21] Halldorsson T. I. Rytter D. Haug L. S. Bech B. H. Danielsen I. Becher G. (2012). Prenatal Exposure to Perfluorooctanoate and Risk of Overweight at 20 Years of Age: A Prospective Cohort Study. Environ. Health Perspect. 120, 668–673. 10.1289/EHP.1104034 22306490 PMC3346773

[B22] Heuvel J. P. V. Kuslikis B. I. Van Rafelghem M. J. Peterson R. E. (1991). Tissue Distribution, Metabolism, and Elimination of Perfluorooctanoic Acid in Male and Female Rats. J. Biochem. Toxicol. 6, 83–92. 10.1002/jbt.2570060202 1941903

[B23] Kalisch-Smith J. I. Simmons D. G. Dickinson H. Moritz K. M. (2017). Review: Sexual Dimorphism in the Formation, Function and Adaptation of the Placenta. Placenta 54, 10–16. 10.1016/J.PLACENTA.2016.12.008 27979377

[B24] Kim S. Cho Y. H. Won S. Ku J.-L. Moon H.-B. Park J. (2019). Maternal Exposures to Persistent Organic Pollutants Are Associated with DNA Methylation of Thyroid Hormone-Related Genes in Placenta Differently by Infant Sex. Environ. Int. 130, 104956. 10.1016/J.ENVINT.2019.104956 31272017

[B25] Krönke A. A. Jurkutat A. Schlingmann M. Poulain T. Nüchter M. Hilbert A. (20212021). Persistent Organic Pollutants in Pregnant Women Potentially Affect Child Development and Thyroid Hormone Status. Pediatr. Res. 91, 690–698. 10.1038/s41390-021-01488-5 PMC8904258 33824444

[B26] Krysiak-Baltyn K. Toppari J. Skakkebaek N. E. Jensen T. S. Virtanen H. E. Schramm K.-W. (2010). Country-specific Chemical Signatures of Persistent Environmental Compounds in Breast Milk. Int. J. Androl. 33, 270–278. 10.1111/J.1365-2605.2009.00996.X 19780864

[B27] Lam J. Koustas E. Sutton P. Johnson P. I. Atchley D. S. Sen S. (2014). The Navigation Guide-Evidence-Based Medicine Meets Environmental Health: Integration of Animal and Human Evidence for PFOA Effects on Fetal Growth. Environ. Health Perspect. 122, 1040–1051. 10.1289/ehp.1307923 24968389 PMC4181930

[B28] Lau C. Butenhoff J. L. Rogers J. M. (2004). The Developmental Toxicity of Perfluoroalkyl Acids and Their Derivatives. Toxicol. Appl. Pharmacol. 198, 231–241. 10.1016/j.taap.2003.11.031 15236955

[B29] Lau C. Thibodeaux J. R. Hanson R. G. Narotsky M. G. Rogers J. M. Lindstrom A. B. (2006). Effects of Perfluorooctanoic Acid Exposure during Pregnancy in the Mouse. Toxicol. Sci. 90, 510–518. 10.1093/toxsci/kfj105 16415327

[B30] Lauritzen H. B. Larose T. L. Øien T. Odland J. Ø. van de Bor M. Jacobsen G. W. (2016). Factors Associated with Maternal Serum Levels of Perfluoroalkyl Substances and Organochlorines: A Descriptive Study of Parous Women in Norway and Sweden. PLoS One 11, e0166127. 10.1371/journal.pone.0166127 27824939 PMC5100957

[B31] Lauritzen H. B. Larose T. L. Øien T. Sandanger T. M. Odland J. Ø. Van De Bor M. (2017). Maternal Serum Levels of Perfluoroalkyl Substances and Organochlorines and Indices of Fetal Growth: a Scandinavian Case-Cohort Study. Pediatr. Res. 81, 33–42. 10.1038/pr.2016.187 27656770 PMC5313514

[B32] Lefebvre T. Fréour T. Ploteau S. Le Bizec B. Antignac J.-P. Cano-Sancho G. (2021). Associations between Human Internal Chemical Exposure to Persistent Organic Pollutants (POPs) and *In Vitro* Fertilization (IVF) Outcomes: Systematic Review and Evidence Map of Human Epidemiological Evidence. Reprod. Toxicol. 105, 184–197. 10.1016/j.reprotox.2021.09.005 34517099

[B33] Leonetti C. Butt C. M. Hoffman K. Hammel S. C. Miranda M. L. Stapleton H. M. (2016). Brominated Flame Retardants in Placental Tissues: Associations with Infant Sex and Thyroid Hormone Endpoints. Environ. Health 15, 199. 10.1186/S12940-016-0199-8 PMC5123327 27884139

[B34] Li Y. Fletcher T. Mucs D. Scott K. Lindh C. H. Tallving P. (2018). Half-lives of PFOS, PFHxS and PFOA after End of Exposure to Contaminated Drinking Water. Occup. Environ. Med. 75, 46–51. 10.1136/oemed-2017-104651 29133598 PMC5749314

[B35] Liew Z. Ritz B. Bonefeld-Jørgensen E. C. Henriksen T. B. Nohr E. A. Bech B. H. (2014). Prenatal Exposure to Perfluoroalkyl Substances and the Risk of Congenital Cerebral Palsy in Children. Am. J. Epidemiol. 180, 574–581. 10.1093/aje/kwu179 25139206 PMC12118604

[B36] Luo D. Pu Y. Tian H. Wu W. Sun X. Zhou T. (2017). Association of In Utero Exposure to Organochlorine Pesticides with Thyroid Hormone Levels in Cord Blood of Newborns. Environ. Pollut. 231, 78–86. 10.1016/J.ENVPOL.2017.07.091 28787707

[B37] Maervoet J. Vermeir G. Covaci A. Van Larebeke N. Koppen G. Schoeters G. (2007). Association of Thyroid Hormone Concentrations with Levels of Organochlorine Compounds in Cord Blood of Neonates. Environ. Health Perspect. 115, 1780–1786. 10.1289/ehp.10486 18087600 PMC2137114

[B38] Maisonet M. Terrell M. L. McGeehin M. A. Christensen K. Y. Holmes A. Calafat A. M. (2012). Maternal Concentrations of Polyfluoroalkyl Compounds during Pregnancy and Fetal and Postnatal Growth in British Girls. Environ. Health Perspect. 120, 1432–1437. 10.1289/ehp.1003096 22935244 PMC3491920

[B39] Mamsen L. S. Björvang R. D. Mucs D. Vinnars M.-T. Papadogiannakis N. Lindh C. H. (2019). Concentrations of Perfluoroalkyl Substances (PFASs) in Human Embryonic and Fetal Organs from First, Second, and Third Trimester Pregnancies. Environ. Int. 124, 482–492. 10.1016/j.envint.2019.01.010 30684806

[B40] Mamsen L. S. Jönsson B. A. G. Lindh C. H. Olesen R. H. Larsen A. Ernst E. (2017). Concentration of Perfluorinated Compounds and Cotinine in Human Foetal Organs, Placenta, and Maternal Plasma. Sci. Total Environ. 596-597, 97–105. 10.1016/j.scitotenv.2017.04.058 28426990

[B41] Martin E. Smeester L. Bommarito P. A. Grace M. R. Boggess K. Kuban K. (2017). Sexual Epigenetic Dimorphism in the Human Placenta: Implications for Susceptibility during the Prenatal Period. Epigenomics 9. 267–278. 10.2217/EPI-2016-0132/EPI-09-267-S1 28234023 PMC5331919

[B42] Mendez M. A. Garcia-Esteban R. Guxens M. Vrijheid M. Kogevinas M. Goñi F. (2011). Prenatal Organochlorine Compound Exposure, Rapid Weight Gain, and Overweight in Infancy. Environ. Health Perspect. 119, 272–278. 10.1289/EHP.1002169 20923745 PMC3040617

[B43] Miller C. N. Dye J. A. Henriquez A. R. Stewart E. J. Lavrich K. S. Carswell G. K. (2020). Ozone-induced Fetal Growth Restriction in Rats Is Associated with Sexually Dimorphic Placental and Fetal Metabolic Adaptation. Mol. Metab. 42, 101094. 10.1016/J.MOLMET.2020.101094 33031959 PMC7588867

[B44] Mustieles V. Arrebola J. P. (2020). How Polluted Is Your Fat? what the Study of Adipose Tissue Can Contribute to Environmental Epidemiology. J. Epidemiol. Community Health 74, 401–407. 10.1136/jech-2019-213181 32019765

[B45] Nakayama S. Harada K. Inoue K. Sasaki K. Seery B. Saito N. (2005). Distributions of Perfluorooctanoic Acid (PFOA) and Perfluorooctane Sulfonate (PFOS) in Japan and Their Toxicities. Environ. Sci. 12, 293 16609670

[B46] Negri E. Metruccio F. Guercio V. Tosti L. Benfenati E. Bonzi R. (2017). Exposure to PFOA and PFOS and Fetal Growth: a Critical Merging of Toxicological and Epidemiological Data. Crit. Rev. Toxicol. 47, 489–515. 10.1080/10408444.2016.1271972 28617200

[B47] Nishimura H. Shiota K. Tanimura T. Matsumoto M. Ueda M. (1977). Levels of Polychlorinated Biphenyls and Organochlorine Insecticides in Human Embryos and Fetuses. Paediatrician 6, 45

[B48] Olsen G. W. Butenhoff J. L. Zobel L. R. (2009). Perfluoroalkyl Chemicals and Human Fetal Development: An Epidemiologic Review with Clinical and Toxicological Perspectives. Reprod. Toxicol. 27, 212–230. 10.1016/j.reprotox.2009.02.001 19429401

[B49] Phuthong S. Reyes-Hernández C. G. Rodríguez-Rodríguez P. Ramiro-Cortijo D. Gil-Ortega M. González-Blázquez R. (2020). Sex Differences in Placental Protein Expression and Efficiency in a Rat Model of Fetal Programming Induced by Maternal Undernutrition. Ijms 22, 237–317. 10.3390/IJMS22010237 PMC7795805 33379399

[B50] Pusiol T. Lavezzi A. Matturri L. Termopoli V. Cappiello A. Piscioli F. (2016). Impact Assessment of Endocrine Disruptors on Sudden Intrauterine and Infant Death Syndromes. Eur. J. Forensic Sci. 3, 8. 10.5455/ejfs.197968

[B51] Rosenfeld C. S. (2015). Sex-specific Placental Responses in Fetal Development. Endocrinology 156, 3422–3434. 10.1210/en.2015-1227 26241064 PMC4588817

[B52] Saoi M. Kennedy K. M. Gohir W. Sloboda D. M. Britz-McKibbin P. (2020). Placental Metabolomics for Assessment of Sex-specific Differences in Fetal Development during Normal Gestation. Sci. Rep. 10, 66222. 10.1038/S41598-020-66222-3 PMC7286906 32523064

[B53] Schecter A. Johnson-Welch S. Tung K. C. Harris T. R. Päpke O. Rosen R. (2006). Polybrominated Diphenyl Ether (PBDE) Levels in Livers of U.S. Human Fetuses and Newborns. J. Toxicol. Environ. Health, Part A 70, 1–6. 10.1080/15287390600748369 17162494

[B54] Sharma A. Mollier J. Brocklesby R. W. K. Caves C. Jayasena C. N. Minhas S. (2020). Endocrine‐disrupting Chemicals and Male Reproductive Health. Reprod. Med. Biol. 19, 243–253. 10.1002/RMB2.12326 32684823 PMC7360961

[B55] Sood R. Zehnder J. L. Druzin M. L. Brown P. O. (2006). Gene Expression Patterns in Human Placenta. Proc. Natl. Acad. Sci. U.S.A. 103, 5478–5483. 10.1073/PNAS.0508035103 16567644 PMC1414632

[B56] Sørli J. B. Låg M. Ekeren L. Perez-Gil J. Haug L. S. Da Silva E. (2020). Per- and Polyfluoroalkyl Substances (PFASs) Modify Lung Surfactant Function and Pro-inflammatory Responses in Human Bronchial Epithelial Cells. Toxicol. Vitro 62, 104656. 10.1016/j.tiv.2019.104656 31536757

[B57] Stockholm Convention (2008b). What Are POPs?. Available at: http://www.pops.int/TheConvention/ThePOPs/AllPOPs/tabid/2509/Default.aspx (Accessed August 15, 2019).

[B58] Stockholm Convention (2008a). Listing of POPs in the Stockholm Convention. Available at: http://www.pops.int/TheConvention/ThePOPs/tabid/673/Default.aspx (Accessed August 15, 2019).

[B59] Tan J. Loganath A. Chong Y. S. Obbard J. P. (2009). Exposure to Persistent Organic Pollutants In Utero and Related Maternal Characteristics on Birth Outcomes: a Multivariate Data Analysis Approach. Chemosphere 74, 428–433. 10.1016/J.CHEMOSPHERE.2008.09.045 18986677

[B60] Tang-Péronard J. L. Andersen H. R. Jensen T. K. Heitmann B. L. (2011). Endocrine-disrupting Chemicals and Obesity Development in Humans: a Review. Obes. Rev. 12, 622–636. 10.1111/J.1467-789X.2011.00871.X 21457182

[B61] Tekola-Ayele F. Workalemahu T. Gorfu G. Shrestha D. Tycko B. Wapner R. (2019). Sex Differences in the Associations of Placental Epigenetic Aging with Fetal Growth. Aging 11, 5412–5432. 10.18632/AGING.102124 31395791 PMC6710059

[B62] Thibodeaux J. R. Hanson R. G. Rogers J. M. Grey B. E. Barbee B. D. Richards J. H. (2003). Exposure to Perfluorooctane Sulfonate during Pregnancy in Rat and Mouse. I: Maternal and Prenatal Evaluations. Toxicol. Sci. 74, 369–381. 10.1093/toxsci/kfg121 12773773

[B63] Toft G. Jönsson B. A. G. Lindh C. H. Giwercman A. Spano M. Heederik D. (2012). Exposure to Perfluorinated Compounds and Human Semen Quality in Arctic and European Populations. Hum. Reprod. 27, 2532–2540. 10.1093/humrep/des185 22647447

[B64] Trasande L. Zoeller R. T. Hass U. Kortenkamp A. Grandjean P. Myers J. P. (2015). Estimating Burden and Disease Costs of Exposure to Endocrine-Disrupting Chemicals in the European Union. J. Clin. Endocrinol. Metabolism 100, 1245–1255. 10.1210/jc.2014-4324 PMC4399291 25742516

[B65] Trudel D. Scheringer M. Von Goetz N. Hungerbühler K. (2011). Total Consumer Exposure to Polybrominated Diphenyl Ethers in North America and Europe. Environ. Sci. Technol. 45, 2391–2397. 10.1021/es1035046 21348481

[B66] Valvi D. Mendez M. A. Garcia-Esteban R. Ballester F. Ibarluzea J. Goñi F. (2014). Prenatal Exposure to Persistent Organic Pollutants and Rapid Weight Gain and Overweight in Infancy. Obesity 22, 488–496. 10.1002/OBY.20603 23963708

[B67] Washino N. Saijo Y. Sasaki S. Kato S. Ban S. Konishi K. (2009). Correlations between Prenatal Exposure to Perfluorinated Chemicals and Reduced Fetal Growth. Environ. Health Perspect. 117, 660–667. 10.1289/EHP.11681 19440508 PMC2679613

[B68] Weinheimer C. Wang H. Comstock J. M. Singh P. Wang Z. Locklear B. A. (2020). Maternal Tobacco Smoke Exposure Causes Sex-Divergent Changes in Placental Lipid Metabolism in the Rat. Reprod. Sci. 27, 631–643. 10.1007/S43032-019-00065-W 32046449 PMC7539808

[B69] Widnes C. Flo K. Acharya G. (2017). Exploring Sexual Dimorphism in Placental Circulation at 22-24 Weeks of Gestation: A Cross-Sectional Observational Study. Placenta 49, 16–22. 10.1016/j.placenta.2016.11.005 28012450

[B70] Yang C. Fang J. Sun X. Zhang W. Li J. Chen X. (2021). Prenatal Exposure to Organochlorine Pesticides and Infant Growth: A Longitudinal Study. Environ. Int. 148, 106374. 10.1016/J.ENVINT.2020.106374 33476788

[B71] Yu P. Chen Y. Ge C. Wang H. (2021). Sexual Dimorphism in Placental Development and its Contribution to Health and Diseases. Crit. Rev. Toxicol. 51, 555–570. 10.1080/10408444.2021.1977237/FORMAT/EPUB 34666604

[B72] Yu W.-G. Liu W. Jin Y.-H. Liu X.-H. Wang F.-Q. Liu L. (2009). Prenatal and Postnatal Impact of Perfluorooctane Sulfonate (PFOS) on Rat Development: a Cross-Foster Study on Chemical Burden and Thyroid Hormone System. Environ. Sci. Technol. 43, 8416–8422. 10.1021/es901602d 19924978

[B73] Zafeiri A. Fowler P. A. (2021). Expression Patterns of Analgesic Metabolising Machinery in 1st and 2nd Trimester Human Fetal Liver and Gonads. J. Endocr. Soc. 5, A488. 10.1210/JENDSO/BVAB048.998

[B74] Zota A. R. Mitro S. D. Robinson J. F. Hamilton E. G. Park J.-S. Parry E. (2018). Polybrominated Diphenyl Ethers (PBDEs) and Hydroxylated PBDE Metabolites (OH-PBDEs) in Maternal and Fetal Tissues, and Associations with Fetal Cytochrome P450 Gene Expression. Environ. Int. 112, 269–278. 10.1016/j.envint.2017.12.030 29316516 PMC6561508

